# Ion Channels in Cardiac Non-Excitable Cells: From Single-Cell Atlases to Disease

**DOI:** 10.3390/cells15191790

**Published:** 2026-09-30

**Authors:** Weixuan Chen, Ashlyn A. Fitzgerald, Brett Bardallis, Anu Priya Bharathi Rajan, Pengfei Liang

**Affiliations:** 1 Department of Physiology & Cell Biology, The Ohio State University Wexner Medical Center, Columbus, OH 43210, USA; 2 Bob and Corrine Frick Center for Heart Failure and Arrhythmia, Dorothy M. Davis Heart and Lung Research Institute, The Ohio State University Wexner Medical Center, Columbus, OH 43210, USA

**Keywords:** cardiac non-myocytes, ion channels, mechanotransduction, cardiac fibrosis, single-cell transcriptomics, heart failure

## Abstract

Cardiovascular disease is shaped not only by cardiomyocyte excitability but also by electrophysiological and membrane-signaling programs in cardiac fibroblasts, endothelial cells, mural cells, immune cells, and adipocytes. Recent single-cell RNA sequencing, spatial transcriptomic, and disease-atlas studies have revealed broad expression of ion-channel transcripts across these non-excitable cardiac cell populations. However, transcript detection alone does not establish functional channel activity, cell-state specificity, or therapeutic relevance. This narrative Review therefore focuses on mechanistically supported channel–cell–disease axes rather than providing an exhaustive catalogue of ion channels detected in cardiac non-myocytes. We synthesize evidence for mechanosensitive channels, transient receptor potential channels, calcium-entry pathways, acid-sensing channels, and potassium, sodium, and chloride channels, as well as connexin hemichannels and pannexin channels. We emphasize studies in which omics-based channel nomination is supported by functional validation through electrophysiology, Ca^2+^ imaging, mechanostimulation, pharmacological intervention, or cell-selective genetic perturbation. Particular attention is given to mechanisms linking mechanical stress, Ca^2+^ entry, membrane potential, cell-volume regulation, ATP release, immune activation, vascular tone, fibrosis, and metabolic dysfunction. We also identify key evidence gaps where expression-based observations require direct functional testing in human tissues and disease-relevant cardiac models. By distinguishing atlas-level association from causal channel function, this Review provides a framework for prioritizing non-myocyte ion-channel mechanisms as therapeutic targets in cardiovascular diseases.

## 1. Introduction

Cardiac non-excitable cells are not passive structural elements surrounding electrically active myocytes. Fibroblasts organize and remodel the extracellular matrix, endothelial cells form the vascular and endocardial interface, pericytes and vascular smooth-muscle cells stabilize and regulate the microcirculation, immune cells coordinate injury responses and resolution, and adipocytes provide metabolic, paracrine, and inflammatory signals. Together, these cell populations define myocardial architecture, vascular tone, immune surveillance, tissue repair, and metabolic homeostasis. Their functions are tightly interdependent with cardiomyocytes: non-myocytes supply mechanical support, extracellular matrix cues, oxygen and nutrient delivery, paracrine signals, immune mediators, and, in some contexts, electrical or electrochemical coupling through gap junctions, hemichannels, extracellular ATP, and Ca^2+^-dependent signaling.

Cardiovascular disease therefore emerges from interactions between cardiomyocytes and a diverse non-myocyte compartment rather than from cardiomyocyte dysfunction alone. Single-cell and spatial transcriptomic studies now show that fibroblasts, endothelial cells, pericytes, vascular smooth-muscle cells, myeloid and lymphoid immune cells, and adipocytes occupy anatomically and disease-specific states within the human heart. These datasets have revealed extensive ion-channel transcripts outside cardiomyocytes, raising a central question for cardiovascular biology: which channels merely mark cell identity or activation state, and which channels actively drive disease mechanisms?

This question is timely because ion-channel biology in cardiovascular medicine has historically focused on cardiomyocyte excitability, conduction, and arrhythmia. In contrast, non-excitable cardiac cells use ion channels to regulate Ca^2+^ entry, membrane potential, cell volume, ATP release, inflammatory activation, matrix production, vascular tone, and intercellular communication. These functions place ion channels at the interface between mechanical load, metabolic stress, immune activation, vascular remodeling, and fibrotic repair. A major challenge, however, is that transcript abundance does not prove channel activity. Membrane localization, electrophysiology, Ca^2+^ imaging, mechanostimulation, conditional genetics, and pharmacological perturbation are required to establish functional relevance.

To preserve focus, this Review does not attempt to cover every channel reported in every non-myocyte population. Instead, it prioritizes mechanisms in which transcriptomic or proteomic nomination can be connected to functional validation in cardiac non-excitable cells or to disease-relevant evidence from vascular, immune, adipose, or other systems. We organize the field around channel–cell–disease mechanisms rather than a catalogue of expression. The Review first considers channel families that link force, Ca^2+^ entry, membrane potential, pH, metabolism, and paracrine communication to non-myocyte state transitions. It then integrates these mechanisms across fibrosis, heart failure (HF), arrhythmogenic remodeling, ischemic injury, vascular dysfunction, atherosclerosis, and metabolic cardiovascular disease. The goal is to provide a framework for moving from single-cell discovery to causal channel–cell–disease maps.

## 2. Scope and Literature-Search Methods

This is a narrative, mechanism-focused review rather than a systematic review. We searched PubMed, Web of Science, and Google Scholar for studies combining terms related to cardiac non-myocytes, non-excitable cells, fibroblasts, endothelial cells, smooth-muscle cells, pericytes, macrophages, lymphocytes, adipocytes, ion channels, calcium channels, mechanosensitive channels, TRP channels, potassium channels, sodium channels, chloride channels, connexins, pannexins, single-cell RNA sequencing, spatial transcriptomics, cardiac fibrosis, myocardial infarction, heart failure, arrhythmia, vascular remodeling, atherosclerosis, and ischemia–reperfusion injury. Priority was given to studies that linked expression or omics evidence to functional assays, genetic or pharmacological perturbation, human cardiac tissue, or disease models. Expression-only findings were included mainly when they nominated testable mechanisms or highlighted gaps requiring functional validation.

## 3. From Single-Cell Discovery to Functional Channel–Cell–Disease Mechanisms

The sections below are organized as mechanistic modules rather than a cell-by-cell inventory. Short evidence fragments are interpreted only when they clarify a recurring mechanism, identify a disease-relevant validation gap, or explain why a channel family is prioritized for future testing.

### 3.1. Mechanosensitive Ion Channels

Every cardiac cycle imposes stretch, compression, tension, and shear on myocardial and vascular cells. Mechanosensitive ion channels convert these forces into ionic flux and downstream biochemical signaling by changing their open probability [1,2,3]. In non-excitable cells, this coupling can alter calcium dynamics, membrane potential, cytoskeletal organization, inflammatory signaling, matrix production, and vascular tone. These channels, therefore, provide a direct molecular interface between hemodynamic load and tissue remodeling.

#### 3.1.1. PIEZO1

The PIEZO family comprises PIEZO1 and PIEZO2. PIEZO2 is enriched in sensory tissues, whereas PIEZO1 is broadly expressed and contributes to cardiovascular development and function. Mechanical forces and osmotic changes activate PIEZO1, leading to calcium influx that can influence proliferation, differentiation, and cell state signaling [4,5,6]. The embryonic lethality and disorganized vasculature caused by Piezo1 loss in mice establish its developmental importance [4,7,8]. Piezo1 is also upregulated through an angiotensin II-ERK1/2 pathway after myocardial infarction (MI) in male Sprague-Dawley rats [9,10]. By contrast, its cell-specific functions in the adult heart remain incompletely resolved.

##### PIEZO1 in Fibroblast Mechanotransduction and Fibrotic Signaling

Information on the responses of cardiac fibroblasts to mechanical forces is limited to in vitro studies due to constraints in experimental parameters. In vitro studies have shown that cardiac fibroblasts can respond to many mechanical forces, including mechanical strain through stretch, cyclic stretch, and compression [10].

**Figure 1:**
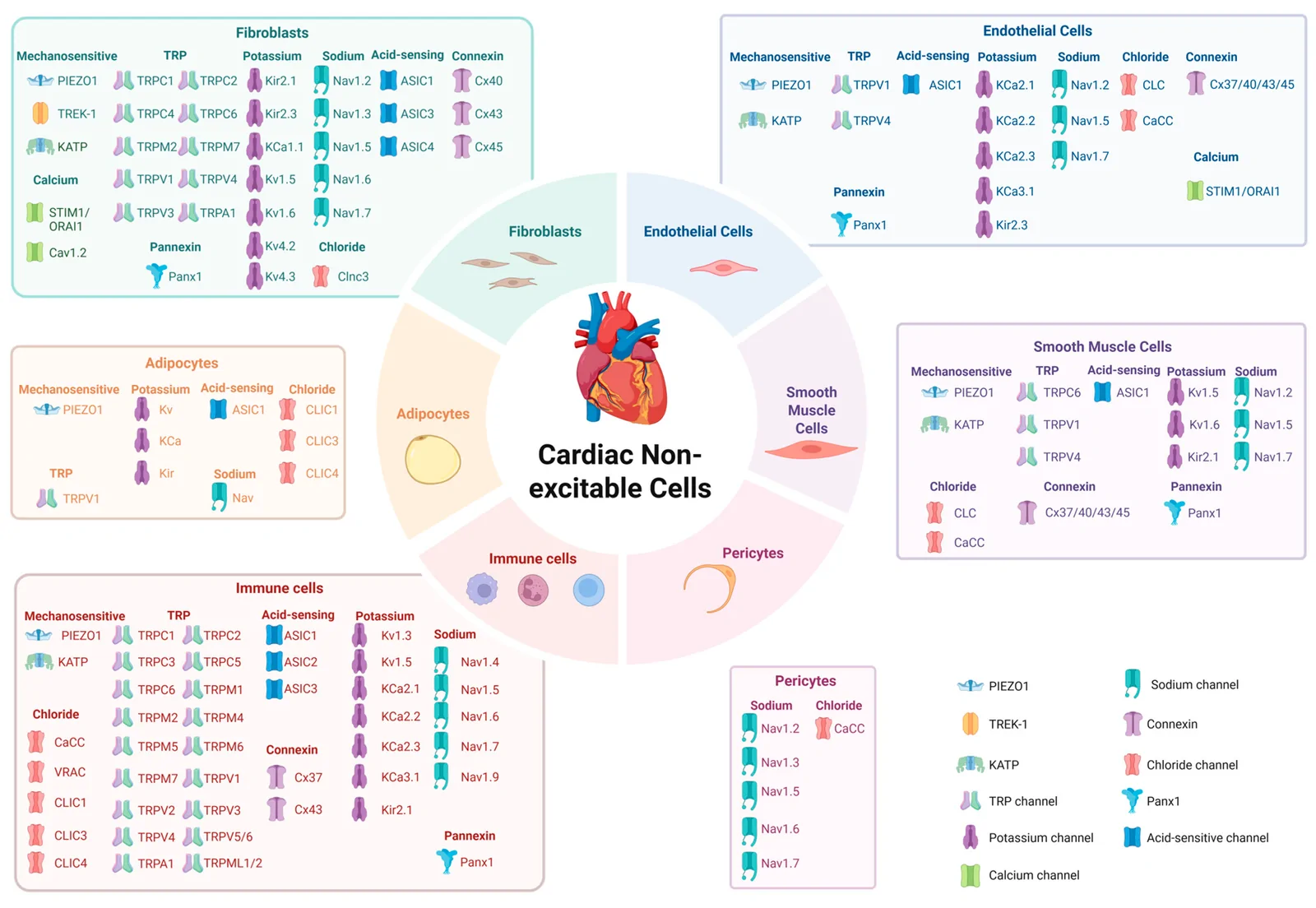
Overview of ion-channel expression in major cardiac non-excitable cells.

PIEZO1 is present in both murine and human cardiac fibroblasts and shows even higher expression than in cardiomyocytes. It has been shown to play a role in cardiac outflow tract and aortic valve development [10,11,12], the response to cyclic stretch [10,13], and mediating homeostatic cardiac mechano-chemical transduction [10,14]. It can also be affected by extracellular matrix (ECM) stiffness and has been linked to atrial fibrillation (AF) [15,16,17,18]. Compared with fibroblasts isolated from patients in sinus rhythm, fibroblasts isolated from patients with AF show increased PIEZO1 expression [10,17]. PIEZO1 activation by Yoda1 was coupled to IL-6 activation, and increased circulating IL-6 is associated with multiple cardiovascular diseases (CVDs), including cardiac fibrosis, hypertension [10,19,20,21], ECM composition alterations, cardiomyocyte hypertrophy [10,22], and altered crosstalk between myocytes and fibroblasts [10,23] (Figure 1).

PIEZO1-mediated Ca^+^ influx can also engage large-conductance Ca^+^-activated K^+^ channel KCa1.1/BK (KCNMA1) activity, illustrating functional channel crosstalk in human fibroblasts [10,17] (Table 1, Figure 1).

##### PIEZO1 in Vascular Mechanotransduction and Endothelial-Smooth Muscle Coupling

PIEZO1 is expressed in endothelial cells in both human and mouse and is involved in several key functions, such as cell adhesion, permeability, proliferation, and migration by activating ADAM10 and ADAM17, which release the ADAM substrates JAM-1 and TNFR1, as well as the ADAM-dependent loss of VE-cadherin at endothelial junctions [4,52]. Structurally, it is required to form and regulate vascular structures by acting as a shear-stress sensor via calcium influx in response to increased fluid flow [8,10,53,54] and as a blood-pressure and baroreceptor-reflex sensor [10,55]. Loss of PIEZO1 in endothelial cells can alter the stress fiber organization and cell orientation in response to shear stress [8,54].

PIEZO1 is also expressed in smooth muscle cells (SMCs), where it contributes to small-artery remodeling during hypertension in mouse models. Together with endothelial PIEZO1, smooth-muscle PIEZO1 supports arterial mechanotransduction [10,54] (Table 2, Figure 1).

##### PIEZO1 in Mechanoinflammatory Immune-Cell Responses

The immune cells can be exposed to various types of mechanical stimulation in the heart. For example, when they enter the tissue from the bloodstream, they are exposed to increased shear stress upon adhesion to the endothelium and mechanical stretch when extravasated into the vessel wall. After entering the tissue, they are exposed to a dynamic environment and changes in various disease stages. For example, hypertension enhances fluid shear stresses and mechanical stretch, and atherosclerosis and fibrosis can increase tissue stiffness [2,87].

Macrophages and monocytes highly express PIEZO1. Macrophages with Piezo1 knockout exhibit reduced inflammatory activation and the transcription factor that promotes inflammation, hypoxia-inducible factor 1 (HIF-1) [2,88,89]. In CVD, PIEZO1 expression on monocytes and macrophages is associated with aortic valve stenosis. Stenosis can increase fluid shear stress on monocytes and macrophages, promoting PIEZO1-mediated Ca^+^ influx, which leads to CD11b activation and cell adhesion. Stenosis can also enhance phagocytosis and oxidized low-density lipoprotein uptake in monocyte-derived macrophages [2]. The expression of PIEZO1 is also linked to the regulation of angiogenesis, depending on the local microenvironment and specific stimuli. Under tissue-reparative conditions, PIEZO1 activation in the macrophages can promote angiogenesis and tissue repair [90,91,92], but under ischemia, the macrophages can inhibit angiogenesis by acting as a pro-inflammatory phenotype through PIEZO1-mediated calcium signaling [91,93]. These findings indicate that PIEZO1 can influence the development and progression of CVD through its expression in myeloid cells, especially monocytes and macrophages.

PIEZO1 is also present in T cells and regulates their function. For example, PIEZO1 deficiency can impair antigen priming of T cells by antigen-presenting cells, and PIEZO1 activation enhances T cell receptor activation [2,94]. Fluid shear stress also enhances T cell activation via PIEZO1 [94,95], and Piezo1-knockout mice have fewer lymphoid progenitor cells [96]. These findings support a role for PIEZO1 in immune regulation. However, limited information is available regarding cardiac-specific lymphoid cells. Future studies are needed to define the functional role of PIEZO1 expression in cardiac lymphoid cells (Table 3, Figure 1).

##### PIEZO1 in Adipocyte Mechanobiology and Metabolic Stress

Adipose tissue disorders are linked to various CVDs due to adipose tissue’s crucial regulatory role in maintaining cardiovascular homeostasis, and adipocytes are mechanosensitive and mechanoresponsive cells. Mechanical stimulation, such as cyclic stretching and vibration, can suppress or promote adipocyte differentiation in progenitor cells and several regulatory signaling pathways, which indicates that mechanical stress is critical in adipogenesis and has an impact on adipocyte fates [133,134]. The alteration of the mechanotransduction signaling pathways has also been shown to regulate inflammation and mitochondrial function [134]. The mechanosensitive ion channels mediate adipocyte differentiation, proliferation, functional maturity, and homeostasis maintenance [134].

PIEZO1 is one of the mechanosensitive ion channels that is highly expressed in adipose tissue, working to regulate adipocyte differentiation, adipose remodeling, and systemic metabolic regulation [6]. Piezo1 deletion in adipocytes is linked with adipose inflammation, and it is more highly expressed in obese mice [6]. In the heart, PIEZO1 is exposed to high levels of cyclical stretch and works to control inflammatory response and lipid metabolism (Table 4, Figure 1).

#### 3.1.2. K2P Channels

Two-pore-domain potassium (K2P) channels provide voltage-independent background potassium conductance and help set resting membrane potential [31]. The family contains six functional groups, including mechanosensitive, acid-sensitive, and alkaline-sensitive channels. TREK-1, TREK-2, and TRAAK are prominent mechanosensitive K2P members [24,31,139]. Their hyperpolarizing currents can counterbalance PIEZO1-mediated depolarization, suggesting that mechanically evoked responses depend on coordinated channel activity rather than a single mechanosensor [3,10,140].

##### K2P Channels in Fibroblast Stretch Responses

Among the mechanosensitive K2P channels, TREK-1 has the clearest functional evidence in cardiac fibroblasts [10,24]. Fibroblast-specific TREK-1 deletion reduced interstitial fibrosis and preserved chamber dimensions and cardiac function after transverse aortic constriction in mice [10,24]. Reduced JNK and c-Jun phosphorylation in TREK-1-deficient fibroblasts further implicates this channel in stretch-, TGF-beta-, and EGF-responsive profibrotic signaling [10,24,141]. These findings support a fibroblast-autonomous role for TREK-1 in pressure-overload remodeling, although the translational consequences of systemic inhibition remain uncertain (Table 1, Figure 1).

#### 3.1.3. KATP Channels

ATP-sensitive potassium (KATP) channels are hetero-octameric complexes that couple cellular energetic state to membrane potential. Intracellular ATP generally closes the channel, whereas declining ATP and increasing ADP favor opening, thereby reducing excitability and energy demand during metabolic stress [10,142]. Mechanical stimulation can also activate KATP currents in atrial myocytes [10,142]. Whether the same mechanosensitivity operates directly in cardiac non-excitable cells requires further study.

##### KATP Channels Couple Fibroblast Energetics to Remodeling

KATP channel components are detected in fibroblasts and increase during myofibroblast differentiation, although functional currents may be limited under basal conditions [10,25,26]. In rats after MI, KATP currents were detected in fibroblasts from the scar’s border zones, supporting disease-dependent channel recruitment [10]. Pharmacological opening reduced myofibroblast maturation and pathological remodeling in human and rodent models [143]. However, systemic KATP activation can shorten cardiomyocyte action potentials and refractory periods, increasing ventricular arrhythmia risk in some ischemia models [10,28,144]. This benefit-risk contrast argues for cell-selective and stage-specific targeting (Table 1, Figure 1).

##### KATP Channels in Coronary Vascular Tone and Metabolic Stress

KATP channels have been shown to be expressed in SMCs and endothelial cells in coronary arteries [10,27]. KATP channels in endothelial cells are recognized for regulating coronary blood flow by mediating vasodilation in response to adenosine [30], hyperosmolarity [29], acidosis [57], and isoflurane [75,145] in the coronary microvasculature [27]. In the coronary endothelium, Kir6.1, Kir6.2, and SUR2 are the major expressed subunits of KATP channels.

In the coronary SMCs, KATP channels have a low unitary conductance and can be blocked by glibenclamide and activated by adenosine [27]. Kir6.1 and SUR2 are the major subunits of the KATP channel that are predominantly expressed [27]. Endogenous vasodilators, such as adenosine [58] and NO [60], have been shown to activate protein kinase A, c-GMP-dependent protein kinase, which in turn activates the Kir6.1 channel; and vasoconstrictors, such as norepinephrine [59], endothelin [146], and angiotensin II [147], can activate protein kinase C to close the Kir6.1 channel. KATP channels are shown to be downregulated in diabetes [61] and hypertension [62,75] (Table 2, Figure 1).

##### KATP Channels in Macrophage Polarization and Inflammatory Restraint

The KATP channel is also shown to be expressed in macrophages. KATP channel activation provides a protective role where it can modulate macrophage polarization by regulating the NF-B signaling pathway, suppressing inflammatory responses, and promoting angiogenesis [91,97] (Table 3, Figure 1).

### 3.2. Transient Receptor Potential (TRP) Channels

TRP channels are tetrameric cation channels encoded by more than 30 genes in seven subfamilies, including TRPC, TRPV, TRPM, TRPP, TRPML, TRPN, and TRPA [3,98,148]. Many TRP channels conduct Ca^2+^ and integrate mechanical, oxidative, thermal, ligand-dependent, and metabolic stimuli [3,98,148]. In cardiac non-excitable cells, the most disease-relevant TRP mechanisms converge on Ca^2+^-dependent transcription, cytoskeletal remodeling, cytokine production, matrix synthesis, endothelial barrier function, vascular tone, and immune-cell activation [3,36,98,103,148,149]. Because individual TRP subfamilies often act across several non-myocyte populations, we discuss them below by channel family and mechanism rather than by repeatedly separating fibroblast, vascular, and immune-cell evidence into short cell-type fragments.

#### 3.2.1. TRPC Channels

Canonical TRP channels (TRPC1-TRPC7) are broadly expressed and include receptor-operated and store-operated Ca^2+^-permeable pathways [46,63,98,127,149]. In fibroblasts, TRPC1, TRPC2, TRPC4, and TRPC6 have been detected in channelome studies, with TRPC6 receiving the strongest mechanistic support [46,63,127,149]. TRPC6 can link TGF-β signaling to p38 MAPK, calcineurin-NFAT activation, myofibroblast differentiation, and profibrotic matrix production [46,63,127]. In vascular smooth-muscle cells, TRPC6 also contributes to pressure sensing and vascular tone, providing a mechanism that is distinct from but potentially complementary to TRPV4-dependent mechanotransduction [63,150]. In lymphocytes, TRPC1, TRPC3, TRPC5, and TRPC6 have been implicated in Ca^2+^ entry, proliferation, activation, or apoptosis [21,98,111]. Thus, TRPC channels should be viewed as Ca^2+^-entry and mechanochemical signaling candidates whose cardiac disease relevance depends strongly on cell type, stimulus, and disease stage.

#### 3.2.2. TRPM Channels

Melastatin TRP channels (TRPM1-TRPM8) include both Ca^2+^-permeable and Ca^2+^-impermeable members [98,151]. TRPM2 and TRPM7 are particularly relevant to cardiac fibroblast biology [18,152,153]. TRPM2 is induced by hypoxic stress in adult rat cardiac fibroblasts, whereas TRPM7 regulates intracellular Ca^2+^ handling, fibroblast proliferation, differentiation, and inflammatory remodeling [18,152,153]. TRPM4, although Ca^2+^-impermeable, can shape membrane potential and thereby indirectly influence Ca^2+^ signaling [98,151]. In post-infarction models, TRPM4 deficiency has been linked to enhanced inflammatory-cell infiltration, macrophage proliferation, and inflammatory gene expression [141]. TRPM2 and TRPM4 are also expressed in T-cell populations and can regulate Ca^2+^-dependent activation, proliferation, and cytokine production [98]. These findings support TRPM channels as regulators of fibroblast stress responses and immune-cell activation, but additional cell-selective cardiac studies are needed to distinguish causal disease mechanisms from stress-associated expression.

#### 3.2.3. TRPV Channels

Vanilloid TRP channels (TRPV1-TRPV6) include non-selective cation channels and highly Ca^2+^-selective epithelial Ca^2+^ channels [36,98,151]. In fibroblasts, TRPV1, TRPV3, and TRPV4 have been linked to proliferation, myofibroblast differentiation, extracellular-matrix production, and pressure-overload or post-infarction remodeling [39,154,155]. TRPV4 has the strongest mechanistic connection to cardiac fibrosis because it couples mechanical stimulation to TGF-β1 signaling and fibroblast activation [154,155]. In endothelial cells, TRPV4 responds to shear stress and other mechanical stimuli, promotes Ca^2+^ entry, and supports endothelium-dependent vasodilation through communication with adjacent vascular smooth-muscle cells [4,136,145,150,155,156,157]. TRPV1 has also been reported in endothelial and smooth-muscle cells, where it can influence nitric oxide signaling, AMP-activated protein kinase pathways, angiogenesis, vasodilation, blood pressure, and metabolic vascular responses [64,69]. In immune cells, TRPV1, TRPV2, TRPV4, TRPV5, and TRPV6 have been associated with macrophage recruitment or phagocytosis, T-cell receptor-induced Ca^2+^ entry, cytokine production, angiogenic signaling, and inflammatory activation [98,103]. Overall, TRPV channels provide a mechanistic bridge between mechanical stress, Ca^2+^ influx, vascular tone, fibrosis, and immune-cell crosstalk.

#### 3.2.4. TRPA1

TRPA1 is the only member of the ankyrin TRP family and is best known as a sensor of environmental irritants, inflammatory pain, and oxidative stress [98]. In cardiovascular non-excitable cells, TRPA1 is emerging as a candidate regulator of fibroblast differentiation and inflammatory crosstalk [46,98]. In fibroblasts, TRPA1 has been linked to TGF-β-sensitive differentiation through calcineurin-dependent activation of nuclear factor of activated T cells (NFAT3) [46]. In cardiovascular macrophages, TRPA1 activation has been associated with dilated cardiomyopathy and pressure-overload-associated fibrotic signaling, whereas TRPA1 inhibition can reduce profibrotic cytokine production [129]. TRPA1 may also modulate TRPV1-dependent CD4^+^ T-cell activation in extracardiac inflammatory models [98]. Direct evidence in cardiac T cells remains limited, but these findings nominate TRPA1 as a testable link between oxidative or inflammatory stress, Ca^2+^-dependent transcription, immune activation, and fibrosis.

#### 3.2.5. Mucolipin Family of the TRP Channels (TRPML)

The TRPML family consists of 3 members, TRPML1-3. TRPML1 is expressed in various tissue types, and it shows high expression in the heart [158]. For example, TRPML1 and TRPML2 were shown to be present in vesicular transport in B cells, but were not reported in T cells [98,159] (Table 3, Figure 1).

#### 3.2.6. Calcium-Entry Channels Beyond TRP

Because Ca^2+^ entry is a central effector of non-myocyte activation, Ca^2+^-selective channels require separate consideration beyond TRP channels. In cardiac fibroblasts, store-operated Ca^2+^ entry (SOCE) mediated by stromal interaction molecule 1 (STIM1) and Orai1 has stronger functional support than classical voltage-gated Ca^2+^ channels. Human and mouse cardiac fibroblasts express STIM1/Orai1, and SOCE can be evoked by store depletion or profibrotic agonists such as angiotensin II and thrombin; in human failing-heart ventricular fibroblasts, ORAI1 protein and Ca^2+^ influx are increased [48,49,50,51]. These data position STIM1/Orai1 as an omics-to-function candidate connecting fibroblast activation, proliferation, migration, collagen production, and heart failure remodeling.

By contrast, voltage-gated Ca^2+^ channel transcripts such as Ca_v_1.2 may be detectable in fibroblast datasets, but functional L-type Ca^2+^ currents are inconsistent or absent in quiescent cardiac fibroblasts, and pharmacological L-type channel modulation does not reliably alter fibroblast Ca^2+^ oscillations [48,148]. Activated myofibroblasts may acquire additional Ca^2+^-entry programs, suggesting that disease state and differentiation status should be tested directly rather than inferred from transcript detection alone.

Endothelial cells and mural cells also use Ca^2+^-entry pathways to regulate vasodilation, permeability, contraction, angiogenesis, and inflammatory recruitment. Endothelial STIM1/Orai1-dependent SOCE and TRPC-linked Ca^2+^ entry cooperate with mechanosensitive TRPV4 and PIEZO1 pathways, while pericyte and smooth-muscle Ca^2+^ entry couples membrane depolarization to vascular tone [4,49,73,75,82,86]. For this Review, these Ca^2+^ channels illustrate the central logic: omics can nominate channel expression, but electrophysiology, Ca^2+^ imaging, and cell-selective perturbation are needed to establish disease-relevant causality.

### 3.3. Acid-Sensing Ion Channels

Acid-sensing ion channels (ASICs) are voltage-independent proton-gated cation channels of the amiloride-sensitive degenerin/epithelial Na^+^ channel superfamily. There are 6 different ASIC isoforms derived from 4 genes (ASIC1-ASIC4) [160]. They are sensors of extracellular protons within pH 4.0–7.0, are highly sensitive to Na^+^, moderately sensitive to Ca^+^, and have low permeability to other monovalent inorganic cations.

ASICs are highly expressed in neurons and detectable in cardiomyocytes [161]. However, their roles in cardiac non-excitable cells remain incompletely defined. Transcriptomic data from adult mouse heart show low ASIC4 and negligible ASIC2 expression in endothelial cells, fibroblasts, and leukocytes. In contrast, ASIC1 and ASIC3 are relatively enriched in these cell types, particularly ASIC3, which is minimally expressed in cardiomyocytes [160,162].

#### 3.3.1. ASICs Link Acidosis to Fibroblast Activation

ASIC1, ASIC3, and ASIC4 are expressed in human cardiac fibroblasts, while ASIC2 is weakly expressed [31]. ASIC activation under acidic or ischemic conditions has been implicated in pro-fibrotic signaling, suggesting a potential role in fibroblast activation during myocardial injury [160] (Table 1, Figure 1).

#### 3.3.2. ASICs in Vascular Tone and Pulmonary Vascular Remodeling

ASIC1 regulates Ca^+^-dependent signaling pathways in vascular SMCs and endothelial cells [74]. In pulmonary arterial SMCs, ASIC1a mediates pulmonary vasoconstriction [40], contributing to vascular remodeling and pulmonary hypertension [74]. In endothelial cells, ASIC1 is associated with vasodilatory signaling and may exert protective effects in pulmonary hypertension [74] (Table 2, Figure 1).

#### 3.3.3. ASICs in Acidic Inflammatory Microenvironments

Direct evidence in cardiac immune cells is limited. However, multiple studies on other systems have been conducted and indicate their important roles. For example, in the central nervous system, ASICs contribute to immune cell function and neuroinflammation.

ASIC1 and ASIC3 are expressed in macrophages, where extracellular acidosis enhances phagocytosis [113] and IL-10 expression, indicating adaptive anti-inflammatory responses [112]. ASIC1 has also been linked to impaired macrophage lipophagy, promoting lipid accumulation and atherosclerosis progression [91,114].

ASIC1-3 are also expressed in dendritic cells and promote IL-12 secretion under acidic conditions, enhancing Th1 responses and T cell activation [112,163]. These findings suggest ASICs link extracellular acidosis to immune activation in cardiovascular inflammation (Table 3, Figure 1).

In addition, ASIC1 is expressed in murine adipocytes, and the systemic knockout of ASIC1a is linked to obesity [136], insulin resistance [136], and hypertension [135]. However, the underlying mechanisms remain unclear (Table 4, Figure 1).

### 3.4. Potassium-Selective Channels

K^+^ channels are widely expressed in various cardiac cell types and represent key determinants of the background leak K^+^ channels that determine the resting potential [118]. Among all these different types of K^+^ channels, the voltage-gated K^+^ channel is the most studied.

#### 3.4.1. Potassium Channels in Fibroblast Membrane Potential and Proliferation

Cardiac fibroblasts express multiple K^+^ channels, including large-conductance Ca^+^-activated K^+^ channels mediated by KCa1.1/BK (KCNMA1), delayed rectifier K^+^ currents represented by Kv1.5 and Kv1.6, transient outward K^+^ currents represented by Kv4.2 and Kv4.3, inward rectifier K^+^ currents represented by Kir2.1 and Kir2.3, and Na^+^ channel isoforms, including Na_v_1.2, Na_v_1.3, Na_v_1.5, Na_v_1.6, and Na_v_1.7 [10].

#### 3.4.2. Potassium Channels in Endothelial Hyperpolarization and Vascular Tone

Arteriolar SMCs express at least four major classes of K^+^ channels, including inward rectifier K^+^ (Kir) channels such as Kir2.1, ATP-sensitive K^+^ (KATP) channels such as Kir6.1-containing channels, Ca^+^-activated K^+^ (KCa) channels, principally KCa1.1/BK, and voltage-gated K^+^ (Kv) channels [75].

These channels regulate membrane potential in SMCs and smooth muscle tone, including blood pressure, blood flow, and microvascular exchange [75]. Kir2.1 mediates K^+^-induced hyperpolarization and vasodilation [75], and is downregulated during hypertension [62]. KCa1.1/BK channels are the dominant KCa channels expressed in SMCs, provide negative feedback to vasoconstriction [164,165], and are implicated in multiple CVDs, including hypertension, hypercholesterolemia, and atherosclerosis [62]. Kv channels, such as Kv1.5 and Kv1.6 [166,167], support the cAMP-protein kinase A signaling pathway for vasodilation [75]. Similar to Kir2.1, Kv channels are also related to hypertension [76] and hyperglycemia [75]. Some SMCs can express small-conductance KCa channels (KCa2.x/SK) [151], but their physiological function is not established.

Arteriolar endothelial cells express KATP, Kv, Kir, and KCa channels [75], especially Kir channels. They accompany the SMCs to contribute to K^+^-induced dilation [78] and hyperpolarization-signal conduction [79]. Kir channels amplify the hyperpolarization initiated by the activation of other K^+^ channels, such as KCa2.x/SK channels (KCa2.1, KCa2.2, and KCa2.3) and KCa3.1/IK channels [168]. These channels can mediate agonist-induced hyperpolarization of endothelial cells and are involved in arteriolar dilation caused by endothelium-dependent vasodilators, such as acetylcholine and histamine [75,77] (Table 2, Figure 1).

#### 3.4.3. Potassium Channels in Immune-Cell Activation, Migration, and Vascular Inflammation

K^+^ channels regulate macrophage differentiation, activation, proliferation, and migration. Evidence is strongest for voltage-gated Kv channels and KCa3.1, while BK and SK channel expression has also been reported during macrophage differentiation [91,117,118,120,169,170].

K^+^ channels in macrophages were first reported in the 1980s. Altering the stoichiometry of the voltage-gated K^+^ channels Kv1.3 and Kv1.5 can change macrophage responses and biophysical properties [18,169,170]. These channels promote macrophage migration by activating the ERK signaling pathway and increasing its expression in acute coronary syndrome [116]. The inwardly rectifying K^+^ channel Kir2.1 and the Ca^+^-activated K^+^ channel KCa3.1 promote a more negative resting membrane potential and contribute to macrophage proliferation and activation [18,118,171], and KCa3.1 inhibition reduces pro-inflammatory gene expression during macrophage polarization [91,120]. Transcriptomic data show that KCNN4, the gene encoding KCa3.1, is highly expressed in the MI border zone and correlates with macrophage recruitment and pro-inflammatory polarization [119]. Together with atherosclerosis studies, these findings identify KCa3.1 as a candidate regulator of macrophage polarization and vascular inflammation [91,120,157].

In T cells, Kv1.3 and KCa3.1 sustain calcium-dependent immune signaling through CRAC channel coupling, enabling NFAT-dependent transcription [172,173]. Kv1.3 channels are enriched in activated effector memory T cells and have emerged as important mediators of autoimmune and chronic inflammatory diseases [174]. KCa3.1 channels similarly regulate T cell activation, motility, and chemotaxis by modulating calcium signaling and cytoskeletal rearrangement [115]. Beyond T cells, potassium channels also contribute to B-cell activation and natural killer cell cytotoxicity, indicating broader immunomodulatory functions within the adaptive immune system.

In the cardiac microenvironment, lymphocyte potassium channels may therefore contribute to inflammatory signaling, immune-cell recruitment, fibrosis, and arrhythmogenic remodeling during myocardial injury and HF (Table 3, Figure 1).

#### 3.4.4. Potassium Channels in Adipocyte Differentiation and Metabolic Homeostasis

Potassium channels are expressed in adipocytes and contribute to adipocyte differentiation, membrane potential regulation, insulin sensitivity, metabolic homeostasis, adipogenesis, and adipocyte maturation [6]. K^+^ influx modulates Ca^+^ entry and downstream metabolic signaling pathways important for lipid storage and thermogenesis.

Dysregulation of adipocyte K^+^ channel activity has been associated with insulin resistance, obesity, altered adipokine secretion, and systemic metabolic dysfunction, all of which contribute to increased cardiovascular risk. However, roles in cardiac adipose tissue and epicardial adipocytes remain unclear and require further investigation (Table 4, Figure 1).

### 3.5. Sodium-Selective Channels

#### 3.5.1. Sodium Channels in Fibroblast Excitability and Intercellular Electrical Coupling

Two types of voltage-gated Na^+^ currents with distinct pharmacological properties have been detected in approximately 61% of cardiac fibroblasts: a tetrodotoxin-sensitive current (TTX) associated with Na_v_1.2, Na_v_1.3, Na_v_1.6, and Na_v_1.7, and a TTX-resistant current associated with Na_v_1.5 [31]. These channels contribute to fibroblast excitability, proliferation, and intercellular electrical communication, although their physiological significance remains incompletely defined (Table 1, Figure 1).

#### 3.5.2. Sodium Channels in Vascular Cell Signaling

Voltage-gated Na^+^ channels are expressed in vascular endothelial cells and SMCs, where they contribute to membrane excitability, vascular tone regulation, cellular migration, and proliferation. Multiple Na_v_ channel isoforms, including Na_v_1.2, Na_v_1.5, and Na_v_1.7, have been reported in vascular SMCs and endothelial cells [80].

In vascular SMCs, Na^+^ influx through voltage-gated Na^+^ channels indirectly promotes Ca^+^ entry through voltage-dependent Ca^+^ channels, thereby regulating vasoconstriction and vascular reactivity. Sodium channel activity has also been linked to SMC proliferation and migration during vascular remodeling and atherosclerosis progression [80].

In endothelial cells, Na^+^ channels may participate in mechanotransduction, endothelial permeability, and nitric oxide signaling, although their precise functional role in the cardiovascular system remains incompletely defined [80].

Evidence for Na^+^ channel expression in cardiac pericytes is limited; however, studies from cerebral and peripheral microvasculature suggest they may influence pericyte contraction and communication with endothelial cells [75]. Additional studies are required to clarify the physiological and pathological roles of Na^+^ channels in cardiac vascular non-myocyte populations (Table 2, Figure 1).

#### 3.5.3. Sodium Channels in Monocyte-Macrophage Inflammation and Lymphocyte Signaling

Voltage-gated Na^+^ channels are expressed in both monocytes and macrophages and regulate immune responses by mediating cellular polarization and proliferation [91,121]. Selective Na_v_1.4 or Na_v_1.9 inhibition alters macrophage polarization dynamics [91,121], and nonselective Na^+^ channel inhibition improves post-infarction left ventricular remodeling in a rat MI reperfusion-injury model [91,175].

Mutation of SCN5A, the gene that encodes the voltage-dependent cardiac sodium channel (Na_v_1.5), in macrophages has been reported to exacerbate the arrhythmic phenotype in co-cultured cardiomyocytes, indicating the impact of the macrophages on cardiomyocyte electrophysiology [91,122].

Voltage-gated Na^+^ channels are increasingly recognized as important regulators of lymphocyte activation, migration, and intracellular signaling despite immune cells being traditionally classified as non-excitable. Multiple Na_v_ channel isoforms, including Na_v_1.5, Na_v_1.6, and Na_v_1.7, have been identified in T lymphocytes and other immune populations, where they contribute to membrane depolarization and Ca^+^-dependent signaling pathways [80]. Sodium influx can modulate intracellular ion homeostasis and amplify signaling cascades required for T cell receptor activation, cytokine production, and immune-cell motility.

Multiple Na_v_ isoforms, including Na_v_1.5, Na_v_1.6 and Na_v_1.7, are expressed in T lymphocytes to regulate membrane depolarization, lymphocyte migration and inflammatory responses [123]. Increasing evidence also links sodium signaling to immunometabolic regulation, with elevated extracellular Na^+^ promoting pro-inflammatory immune activation through SGK1-dependent pathways [176]. These findings suggest that sodium-dependent immune signaling may contribute to hypertension, vascular inflammation, and adverse cardiac remodeling. However, the precise roles of Na_v_ channel isoforms in cardiac lymphoid populations remain incompletely characterized (Table 3, Figure 1).

#### 3.5.4. Sodium Signaling in Adipocyte Dysfunction and Metabolic Cardiovascular Risk

Voltage-gated Na^+^ channels have been identified in adipocytes and adipose-derived stem cells, where they regulate membrane potential, adipogenesis, and intracellular ion homeostasis [6].

Altered Na^+^ signaling has been associated with obesity, insulin resistance, and metabolic dysfunction, suggesting a potential contribution to cardiovascular disease. However, the specific Na_v_ isoforms and functional roles in cardiac adipose tissue remain poorly characterized (Table 4, Figure 1).

### 3.6. Chloride Channels

#### 3.6.1. Chloride Channels in Fibroblast Proliferation and Volume Regulation

Chloride current (ICl) is expressed in 7% of fibroblasts and represented by Clnc3 channels [31]. It was shown in human cardiac fibroblasts that the chloride channel ICl.vol is involved in cardiac fibroblast proliferation by promoting cell cycle progression through mediating the cyclin D1 and cyclin E expression [41] (Table 1, Figure 1).

#### 3.6.2. Chloride Channels in Endothelial and Mural-Cell Contractile Signaling

Arteriolar SMCs and endothelial cells express one or more types of chloride (Cl^−^) channels, including Ca^+^-activated Cl^−^ channels (CaCCs), encoded by TMEM16A, and the CLC chloride channel family [75,177]. Activation of Cl^−^ channels promotes Cl^−^ efflux, leading to membrane depolarization and activation of L-type Ca^+^ channels, thereby increasing intracellular Ca^+^ concentration and SMC contraction [81,82].

CLC-3 is one member of the CLC chloride channel family and is expressed in the human heart, especially the cardiac vascular SMCs, for cell volume homeostasis, cell proliferation, and apoptosis, and has been linked to hypertension-associated vascular disease [83].

Similar to the SMCs, Cl^−^ currents, specifically CaCCs, can be activated in pericytes in response to contractile agonists, leading to membrane depolarization and regulating local hemodynamics [81,82] (Table 2, Figure 1).

#### 3.6.3. Chloride Channels in Immune-Cell Migration and Phagosomal Function

The expression of chloride (Cl^−^) channels has not been reported in cardiac macrophages, but it has been reported in macrophages from other systems and indicates a potential role in the heart. For example, CLIC1 was reported in macrophages and linked to secretion of proinflammatory molecules in chronic inflammatory diseases [91]. CLIC3 was shown to promote macrophage polarization via the NF-B signaling pathways, and its inhibition was shown to be anti-inflammatory [91,126]. Moreover, CLIC4-knockout and CLIC4-overexpressing macrophages were associated with high and low expression of proinflammatory activators, such as IL-1 and iNOS, respectively [91,127].

Cl^−^ channels are essential regulators of lymphocyte membrane potential, cell volume homeostasis, proliferation, migration, and activation. Multiple chloride channel families, including volume-regulated anion channels (VRACs), chloride intracellular channels (CLICs), cystic fibrosis transmembrane conductance regulator (CFTR), and Ca^+^-activated chloride channels, are expressed in T lymphocytes and other immune cells [124]. Chloride flux contributes to the maintenance of the electrochemical gradient required for sustained calcium influx during T cell receptor activation and cytokine secretion.

VRACs are particularly important during lymphocyte activation because rapid cell volume changes are required for proliferation, migration, and immune synapse formation [125]. CLIC proteins additionally regulate oxidative stress responses, inflammasome signaling, and macrophage inflammatory activation [91,124]. CLIC1 and CLIC4 have been implicated in reactive oxygen species production and pro-inflammatory cytokine secretion, linking chloride channel activity to immune-cell inflammatory phenotypes [91,124].

Chloride channels also contribute to immune-cell migration through regulation of cytoskeletal dynamics and osmotic balance during chemotaxis. Dysregulated chloride signaling has been associated with chronic inflammation, autoimmune disease, and impaired immune-cell function [138]. In the cardiac microenvironment, chloride channels may therefore influence inflammatory signaling, immune-cell infiltration, and fibro-inflammatory remodeling following myocardial injury. However, direct studies investigating chloride channel function in cardiac lymphoid populations remain limited (Table 3, Figure 1).

#### 3.6.4. Chloride Channels in Adipocyte Inflammation and Metabolic Dysfunction

Chloride channels are expressed in adipocytes and participate in adipogenesis, cell volume regulation, lipid metabolism, and insulin signaling. Volume-regulated anion channels and CLIC family proteins contribute to osmotic balance and membrane potential regulation during adipocyte differentiation and lipid accumulation [125,137]. Chloride flux also influences intracellular signaling pathways that regulate adipokine secretion and metabolic activity.

Altered Cl^−^ channel activity has been associated with obesity, adipose tissue inflammation, and insulin resistance. Dysregulated chloride homeostasis can impair adipocyte function and contribute to oxidative stress and chronic low-grade inflammation, thereby increasing cardiovascular risk [138]. Although limited studies have specifically examined chloride channels in cardiac adipose tissue, these channels may contribute to adipocyte-mediated inflammatory crosstalk and pathological cardiac remodeling during obesity-associated cardiovascular disease (Table 4, Figure 1).

### 3.7. Connexin Hemichannels and Pannexin Channels

Gap junctions or connexin (Cx) channels are shown to be present between cardiomyocytes, between cardiomyocytes and other cardiac cell types, including fibroblasts, endothelial cells, and macrophages, and also between non-cardiomyocytes [31,75]. There are six connexins expressed in the cardiovascular system, while four of them are highly expressed: Cx37, Cx40, Cx43, and Cx45 [128,152]. Cx43 is the most expressed connexin isoform in the heart [43].

Pannexins (Panx) comprise three subfamilies: Panx1, Panx2, and Panx3. Panx1 is the most widely expressed isoform across the heart and has been linked to immune-cell recruitment, cardiac fibroblast activation, and metabolic changes [43,47]. Unlike connexin channels, which form intercellular gap-junction channels, Panx proteins primarily form membrane channels that support communication between the cytosol and the extracellular environment [43]. Panx3 is expressed at comparatively low levels in the heart, mainly in vascular endothelial cells, but shows significantly increased expression in dilated cardiomyopathy compared with healthy hearts [47]. Panx1 is the most abundant Panx isoform in the heart [43]. Single-cell transcriptomics, Human Protein Atlas data, Western blotting, immunofluorescence, and flow cytometry in human and animal heart models indicate that Panx1 is the major pannexin in fibroblasts and is also expressed in other non-myocyte cell types [47,178].

The activation or opening of the Cx and Panx channels impacts intracellular ionic homeostasis and redox status and facilitates proinflammatory and pro-fibrotic processes via purinergic signaling [43].

#### 3.7.1. Connexin Hemichannels and Pannexin Channels in Fibroblast Activation

Cardiac fibroblasts can couple with the adjacent fibroblasts and myocytes via connexins to mediate the cellular release of ATP and electrical conduction [46,179]. Cx43, Cx45, and Cx40 are shown to be expressed in cardiac fibroblasts. Cx43 is expressed mainly within the gap junctions, Cx45 is activated mainly between fibroblasts and cardiomyocytes, and Cx40 is mainly activated between the two fibroblasts [179]. Among the connexins, Cx43 and Cx45 are the most highly expressed subtypes in cardiac fibroblasts [46]. Cx43 expression has been reported to be increased in activated fibroblasts during fibrogenesis to promote electrical coupling with cardiomyocytes and fibroblasts, leading to the remodeling of cardiac conduction and contraction [42,43,44,45].

Panx1 is highly expressed in cardiac fibroblasts and contributes to fibroblast activation. Mechanical stress, such as osmotic pressure, can stimulate cardiac fibroblasts to release ATP, which can act as a self-activating purinergic signal that activates fibroblasts into myofibroblasts [46,47]. During cellular stress, such as myocardial ischemia, increased ATP release from adjacent cardiomyocytes via Panx1 can also facilitate early fibroblast activation [43,180]. Conversely, Panx1 inhibition can reduce cardiac fibroblast activation by limiting ATP release, producing a cardioprotective effect in experimental models [43,47] (Table 1, Figure 1).

#### 3.7.2. Connexin and Pannexin Channels in Vascular Communication

Endothelial cells are electrically coupled to one another by gap junctions to transmit the cell membrane potential along the endothelium [75]. Endothelial cells are also coupled with SMCs via myoendothelial gap junctions, modulating arteriolar tone and causing vasodilation [75,84,85].

Panx1 is expressed in endothelial cells and contributes to the chronic ischemia–reperfusion (I/R) response that leads to heart failure (HF) [47]. No further decrease in cardiac function was observed in endothelial cell-specific Panx1-knockout mice. Reduced recruitment of CD45^+^ pro-inflammatory macrophages in these mice demonstrates that endothelial Panx1 normally promotes immune-cell infiltration, thereby driving chronic deterioration of cardiac function [47].

Connexins and Panx channels are also expressed in SMCs to regulate cellular function, vessel diameter, and blood flow [43,181]. They specifically control the SMC Ca^+^ dynamics and contractility by facilitating Ca^+^ entry, ATP release, and purinergic signaling [43,182] (Table 2, Figure 1).

#### 3.7.3. Connexin and Pannexin Channels in Immune-Cell Crosstalk

The cardiac resident macrophages in the atrioventricular node highly express Cx43 to form gap junctions with the adjacent cardiomyocytes, enabling synchronous depolarization of the negative resting membrane potential to promote signal propagation. Moreover, Cx43 deletion or macrophage deficiency leads to AV conduction delay [91,130]. However, the gap junctions between the macrophages and cardiomyocytes can also increase the heterogeneity in myocardial repolarization, which increases the risk of ventricular arrhythmias post-MI [91]. This shows the regulatory role of macrophage Cx43 on conduction.

Cx37 in monocytes and macrophages was reported to be atheroprotective by regulating leukocyte recruitment and adhesion to the endothelium. In an atherosclerotic mouse model, the Cx37 deletion has been shown to promote endothelial macrophage adhesion in atherosclerotic plaques via autocrine ATP signaling [128,129].

Cx and Panx channels also play a role in regulating immune-cell activation and inflammatory signaling in lymphoid populations. Cx43 is the predominant connexin isoform expressed in T lymphocytes and antigen-presenting cells, where it contributes to immune synapse formation, calcium wave propagation, and cytokine signaling between immune cells [131]. Gap junction-mediated communication enables the transfer of ions, ATP, cAMP, and other small signaling molecules, thereby coordinating immune-cell activation and adaptive immune responses.

Cx43-mediated signaling has been shown to regulate T cell proliferation, dendritic cell antigen presentation, and lymphocyte migration [131].

Panx1 channels are also expressed in immune cells and mediate ATP release in response to mechanical stress, membrane depolarization, and inflammatory stimulation [132,183]. Extracellular ATP released through Panx1 channels activates purinergic receptors such as P2X7, thereby promoting inflammasome activation, cytokine secretion, and immune-cell recruitment [91,132,183]. Panx1-mediated signaling has further been implicated in T cell migration, macrophage activation, and regulation of adaptive immune responses [132].

Within the heart, Cx- and Panx-mediated signaling may facilitate inflammatory crosstalk between lymphocytes, macrophages, fibroblasts, endothelial cells, and cardiomyocytes during myocardial injury and HF. These pathways may contribute to fibrosis, arrhythmogenesis, and adverse cardiac remodeling, although direct mechanistic studies in cardiac lymphoid populations remain limited (Table 3, Figure 1).

## 4. Integrated Disease Mechanisms in Cardiac Non-Excitable Cells

The cell-type evidence converges on recurrent cardiovascular disease processes, but the therapeutic meaning of a channel depends strongly on cellular context. The same channel family may be protective, maladaptive, or merely associated with disease according to cell identity, mechanical load, inflammatory state, metabolic stress, and disease stage. We therefore group the evidence by major disease category while distinguishing mechanistic cardiac studies from findings extrapolated from vascular, immune, adipose, or other systems.

**Figure 2:**
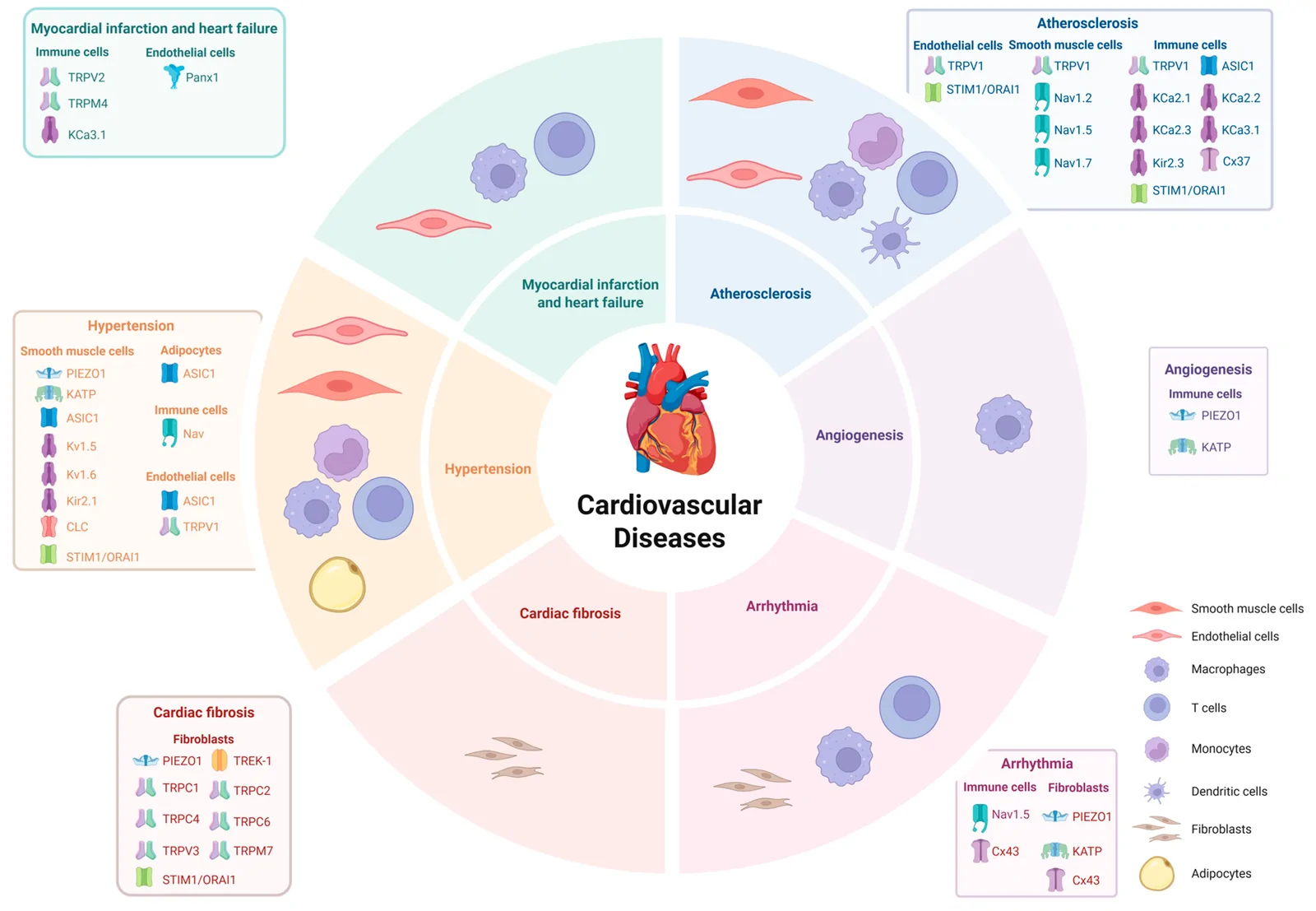
Disease-centered visualization of ion-channel involvement in cardiac non-excitable cells.

This disease-centered synthesis is intended as a prioritization framework rather than a complete ranking of all detected channels. It separates intervention-level support from expression-only evidence and highlights where apparently similar channel effects arise from different cell populations (Table 5, Figure 2).

Not all channels summarized above are equally mature as therapeutic candidates. We therefore prioritize axes that meet at least one of three criteria: direct activity or perturbation data in cardiac non-myocytes, causal genetic or pharmacological evidence in cardiovascular disease models, or sufficient tractability to support cell-selective testing. On this basis, TRPV4, KATP, ASIC1a, Panx1, KCa3.1, sodium-channel pathways, selected Ca^2+^-entry pathways including STIM1/Orai1-dependent store-operated Ca^2+^ entry, and selected metabolic or channel-adjacent axes emerge as near-term or intermediate-term priorities, whereas less-validated transcriptomic candidates are discussed as future directions.

Genetic and pharmacological perturbations provide the strongest support for this framework. In fibrosis and adverse remodeling, TRPV4 deletion after MI and antagonist treatment in diabetic cardiac fibrosis reduced disease severity [154,184], whereas KATP activation with nicorandil limited angiotensin II-stimulated cardiac-fibroblast proliferation [185]. Ca^2+^-entry pathways fit the same disease-centered logic: STIM1/Orai1-dependent store-operated Ca^2+^ entry has been linked to fibroblast activation, endothelial and vascular remodeling, and immune-cell activation, but should be interpreted as one testable Ca^2+^-entry mechanism among several rather than as a separate dominant disease axis [4,48,49,51,73,75,86]. In ischemia–reperfusion injury, ASIC1a inhibition [160], sodium-channel inhibition in monocyte-macrophage responses [175], and Panx1 deletion or nanobody blockade improved outcomes [47,181,193]. These examples show that selected non-myocyte channel pathways can modify disease rather than simply mark injured tissue.

Cell-selective genetic evidence helps distinguish candidate therapeutic targets from broadly expressed channels. Endothelial TRPV4 deletion protects the pressure-overloaded heart [191], partial adeno-associated virus 9 (AAV9)-shCD36 knockdown limits pressure-overload-associated lipotoxicity and dysfunction [192], and KCa3.1 blockade or silencing reduces atherosclerosis in Apoe-null models [157]. Conversely, KATP opening illustrates a translational hazard: activation can protect during ischemia [142,143], yet promote arrhythmia by altering cardiomyocyte repolarization [10,28,144]. Therapeutic development should therefore prioritize cell-selective delivery, disease-stage timing, and explicit assessment of protective channel functions.

## 5. Future Directions

### 5.1. Cell-Specific Mechanisms and Precision Therapeutics

Future studies should prioritize disease-stage-specific, cell-selective mechanisms and direct functional validation. Single-cell and spatial transcriptomics can nominate disease-associated channel programs, but electrophysiology, calcium imaging, controlled mechanostimulation, conditional perturbation, and human tissue studies are required to establish activity and causality. A particularly informative goal is to determine whether channel function identifies reversible states, including activated fibroblasts, inflammatory macrophages, dysfunctional endothelium, contractile-to-synthetic smooth-muscle transitions, and inflamed epicardial adipocytes.

Translation requires caution because most channel families are shared across organs and cell types. Systemic modulation may therefore disrupt protective electrical, vascular, immune, or metabolic functions. Cell-targeted delivery, disease-stage-specific dosing, and combinations with antifibrotic, anti-inflammatory, vascular, or metabolic therapies may improve therapeutic windows. Prioritization should favor channel–cell–disease axes supported by both causal genetics and selective pharmacology.

A practical translational roadmap should prioritize axes with convergent intervention-level evidence: TRPV4 in fibrotic and pressure-overload remodeling; ASIC1a and Panx1 in ischemia–reperfusion injury; KCa3.1 and sodium channels in macrophage-driven vascular inflammation; and CD36-linked metabolic pathways in cardiovascular remodeling [56,121,154,157,160,184,191,192,193]. The decisive experiments must test whether selective modulation in fibroblasts, endothelial cells, macrophages, smooth-muscle cells, or adipocytes improves human-relevant phenotypes without compromising channel functions in cardiomyocytes or other organs.

Because multiple channel families can converge on the same disease phenotype, future therapeutic development should also test channel hierarchy and crosstalk rather than assume that a broad cocktail will be beneficial. Mechanosensitive Ca^2+^-entry channels may sit upstream of K^+^, Cl^−^, pannexin, connexin, inflammasome, or lipid-asymmetry pathways, whereas downstream channels may amplify cell activation, extracellular-vesicle release, ATP signaling, or immune recruitment. Mapping these upstream-downstream relationships with time-resolved perturbation, dual-channel inhibition or activation, and cell-specific genetic models could identify nodal regulators that are more efficient and safer targets than empiric multi-channel combinations. Rational combination therapy may still be useful, but it should be built from defined channel networks, disease stage, and cell type rather than from parallel inhibition of every channel associated with a disease.

A practical route toward causal channel–cell–disease maps should proceed in stages. First, single-cell and spatial atlases can identify candidate channel–cell axes that are enriched in disease-associated fibroblasts, endothelial cells, macrophages, smooth-muscle cells, or adipocytes. Second, these candidates must be tested for activity rather than inferred from transcript abundance, using patch clamp, Ca^2+^ imaging, voltage- or ion-sensitive reporters, mechanostimulation, lipid-transport assays, extracellular-vesicle profiling, and selective pharmacology. Third, causality should be established by cell-selective perturbation in human cardiac slices, engineered multicellular cardiac tissues, organoids, co-culture systems, or disease models of myocardial infarction, pressure overload, ischemia–reperfusion injury, and metabolic stress. For example, TRPV4 can be tested as a fibroblast/endothelial remodeling node, Orai1/STIM1 as a fibroblast Ca^2+^-entry pathway, ASIC1a and Panx1 as ischemia–reperfusion injury mediators, KCa3.1 and sodium-channel pathways as macrophage-inflammatory regulators, and PIEZO1/TRPV4 as upstream mechanosensitive channels that may couple mechanical load to Ca^2+^ entry, extracellular-vesicle release, lipid asymmetry, and fibrotic or vascular remodeling. This staged approach would help distinguish disease-driving channel mechanisms from cell-state markers and prioritize targets with a realistic therapeutic window.

### 5.2. Emerging Ion-Channel Candidates and Membrane Lipid Asymmetry

A second future direction is to move beyond the best-studied channel families and test newer or less-validated channel candidates emerging from cardiac atlases. Examples include TRPM2 in hypoxic fibroblast stress responses, TRPM7 in fibroblast proliferation and inflammatory remodeling, ANO1/TMEM16A and CaCCs in vascular smooth-muscle and pericyte contractility, LRRC8A/VRAC and CLICs in immune-cell and adipocyte volume regulation, and Panx1-mediated ATP release in fibroblast, endothelial, and immune-cell crosstalk [35,47,82,124,125,137,187,194,195]. These candidates should be treated as mechanistic hypotheses rather than expression markers until cell-specific currents, gating stimuli, downstream signaling, and disease-stage causality are established.

Within this emerging space, a more precise lipid-related direction is not lipid transport in general, but the control of membrane lipid asymmetry. P4-ATPase flippases maintain phosphatidylserine and other aminophospholipids in the inner leaflet, whereas scramblases can collapse this asymmetry during calcium elevation, apoptosis, mechanical stress, or inflammatory activation [194,196,197,198,199,200]. This balance provides a plausible membrane-remodeling layer through which ion-channel activity may influence coagulation, extracellular-vesicle release, efferocytosis, angiogenesis, and fibrosis [199,200,201].

TMEM16F/ANO6 is particularly relevant to an ion-channel-focused Review because it is a calcium-activated phospholipid scramblase with channel-like conductance properties [194,196,199,200,202,203]. Direct cardiac evidence remains sparse, but human cardiac fibroblast transcriptomic data and endothelial functional studies support this axis as a testable mechanism linking calcium entry, phosphatidylserine exposure, angiogenic signaling, clot formation, and vascular injury responses [149,197,199,200,201,202,203].

## 6. Conclusions

Cardiac non-excitable cells are active regulators of cardiovascular physiology and disease. Modern single-cell and spatial approaches have made their diversity visible, but the field now needs to determine which ion-channel programs are causal, which are adaptive, and which merely mark cell state. This Review proposes a disease-centered framework in which non-myocyte ion channels operate as multicellular sensors linking mechanical load, membrane potential, calcium dynamics, inflammation, metabolism, vascular tone, and intercellular communication.

A mechanistic view also explains why the same channel family may have divergent effects across contexts. PIEZO1, TRPV4, K2P and KATP channels, potassium, sodium and chloride channels, connexin hemichannels, and pannexin channels can regulate fibroblast activation, endothelial shear responses, macrophage polarization, smooth-muscle tone, adipocyte function, and non-myocyte–cardiomyocyte communication. Expression alone is therefore insufficient for target nomination; the critical questions are whether channel activity is causal or compensatory, which disease stage and cell state mediate the effect, and whether intervention can be achieved without disrupting protective functions in cardiomyocytes or other organs.

The strongest translational opportunity lies in moving from transcriptomic nomination to causal channel–cell–disease maps. Integrating human atlases with electrophysiology, calcium imaging, mechanostimulation, lipidomics, disease models, and cell-selective perturbation will distinguish biomarkers from disease-driving regulators. For experimental cardiology, the field’s next advance will not be a longer expression catalog, but a prioritized framework for testing non-myocyte ion channels as mechanisms and therapeutic targets in human-relevant cardiovascular disease.

## Figures and Tables

**Table 1:** Summary of ion channel expression in fibroblasts. NE, not established.

Type	Name	Function	Disease
Mechanosensitive	PIEZO1	Responds to cyclic stretch and mediates cardiac mechano-chemical transduction	Atrial fibroblast activation/atrial fibrillation-associated remodeling; cardiac fibrosis and ECM remodeling [15,16,17,18,20,21]
	TREK-1	Mechanosensitive K2P conductance linked to fibroblast activation, cardiac fibrosis, and diastolic dysfunction [10,24]	Pressure-overload hypertrophy, fibrosis, and diastolic dysfunction [10,24]
	K_ATP_	KATP subunits/currents are reported in fibroblasts; basal functional activity appears condition-dependent [10,25,26,27]	Modulates myocyte electrophysiology in infarcted hearts; KATP opening may be anti-fibrotic but can be proarrhythmic [10,26,27,28,29,30]
TRP	TRPC1, TRPC2, TRPC4	Detected in fibroblast/channelome studies; may regulate Ca^+^ influx, but channel-specific functions remain incompletely defined [31,32]	Cardiac fibrosis [10]
	TRPC6	Promotes fibroblast differentiation into myofibroblasts [31,33,34]	Cardiac fibrosis [10]
	TRPM2	Hypoxic stress induces TRPM2 expression in adult rat cardiac fibroblasts [35]	Hypoxia-associated fibroblast stress response; direct causal disease role NE [35]
	TRPM7	Regulates Ca^+^ levels, fibroblast proliferation and differentiation [36]	Fibrosis, hypertrophy, and inflammation [37]
	TRPV1	Protective effect on cardiac remodeling by inhibiting fibroblast functions [10]	Protective effects in cardiac remodeling/fibrosis models; direct fibroblast-specific disease role remains limited [10,38]
	TRPV3	Involved in fibroblast proliferation [39]	Cardiac fibrosis [39]
	TRPV4	Couples mechanical stimulation to TGF-beta1 signaling in fibroblasts [31]	Cardiac remodeling [31]
	TRPA1	Mediating fibroblast differentiation [31]	NE
Acid-sensing	ASIC1, ASIC3, ASIC4	Release fibrotic signaling molecules [40]	Hypoxia and ischemic events [40]
Cation-selective: K^+^	Kv1.5, Kv1.6, Kv4.2, Kv4.3, KCa1.1, Kir2.1, Kir2.3	Detected in cardiac fibroblasts; regulate membrane potential/proliferation, but channel-specific functions require validation [31,41]	Fibrosis/remodeling relevance is plausible but direct channel-specific causality remains NE [31,41]
Cation-selective: Na^+^	Na_v_1.2, Na_v_1.3, Na_v_1.5, Na_v_1.6, Na_v_1.7	Voltage-gated Na^+^ currents are detected in cultured human cardiac fibroblasts and may influence fibroblast excitability/electrical coupling [31,41]	Conduction/remodeling relevance is proposed, but direct causal disease evidence remains NE [31,41,42]
Chloride	Clnc3	Cardiac fibroblast proliferation [31,41]	Fibroblast proliferation/remodeling; direct in vivo disease causality remains limited [31,41]
Connexin	Cx43	Activated during fibrogenesis to promote electrical coupling [42,43,44,45]	Cardiac conduction and contraction remodeling [42,43,44,45]
Pannexin	Panx1	Fibroblast activation [46,47]	ATP/P2Y2-mediated profibrotic signaling and emerging cardiac disease relevance [46,47]
Calcium entry	Orai1/STIM1	Store-operated Ca^2+^ entry supports calcineurin-NFAT signaling, fibroblast proliferation, myofibroblast differentiation, and matrix remodeling [48,49,50,51].	Fibrosis and adverse remodeling; direct cell-selective cardiac causality requires further validation [48,51].

**Table 2:** Summary of ion channel expression in endothelial cells, smooth muscle cells, and pericytes. NE, not established.

		Endothelial Cell			Smooth Muscle Cell			Pericytes		
Type	Name	Expression	Function	Disease	Expression	Function	Disease	Expression	Function	Disease
Mechanosensitive	PIEZO1	Expressed	Regulates vascular structure, cell adhesion, permeability, proliferation, and migration [8,52,53,54]	Endothelial inflammation/atherosclerosis in disturbed-flow models; direct cardiac microvascular disease role needs validation [56]	Expressed	Small arteries remodeling [54]	Hypertension [54]	NE	NE	NE
	K_ATP_	Expressed	Regulates coronary blood flow by mediating vasodilation [27,29,30,57]	NE	Expressed	Regulating vasodilation and vasoconstriction [58,59,60]	Diabetes and hypertension [61,62]	NE	NE	NE
TRP	TRPC6	NE	NE	NE	Expressed	Sensing intravascular pressure [63]	NE	NE	NE	NE
	TRPV1	Expressed	Regulates NO signaling, angiogenesis, vasodilation, blood pressure, and metabolic vascular responses [64,65,66,67,68]	Atherosclerosis [69], hypertension [66]	Expressed	Modulates SMC proliferation, migration, apoptosis, and vascular tone [67,68,70,71]	Atherosclerosis [67,68,70,72]	NE	NE	NE
	TRPV4	Expressed	Regulates endothelium-dependent vasodilation and mechanosensitive ADAM10/17 signaling [3,4,10,73]	Vascular remodeling and context-dependent vascular dysfunction [4,73]	Expressed	Vasoconstriction and elevates arterial pressure [63]	NE	NE	NE	NE
Acid-sensing	ASIC1	Expressed	Contributing to endothelial-dependent vasodilation [74]	Pulmonary vascular remodeling/hypertension; endothelial contribution remains incompletely resolved [74]	Expressed	Mediates vasoconstriction [40]	Vascular remodeling and pulmonary hypertension [74]	NE	NE	NE
Cation selective: K^+^	Kv1.5, Kv1.6	NE	NE	NE	Expressed	Regulating vasodilation [75]	Hypertension and hyperglycaemia [75,76]	NE	NE	NE
	KCa2.1/SK1, KCa2.2/SK2, KCa2.3/SK3, KCa3.1/IK	Expressed	Mediate agonist-induced hyperpolarization of endothelial cells and are involved in arteriolar dilation [75,77]	NE	NE	NE	NE	NE	NE	NE
	Kir2.1	Expressed	Contributes to K^+^-induced dilation [78] and the hyperpolarization signal conduction [79]	NE	Expressed	Acts as a sensor for increased extracellular K^+^ for membrane hyperpolarization and vasodilation [75]	Hypertension [62]	NE	NE	NE
	Kir2.3	Expressed	Contributes to K^+^-induced dilation [78] and the hyperpolarization signal conduction [79]	NE	NE	NE	NE	NE	NE	NE
Cation selective: Na^+^	Na_v_1.2, Na_v_1.5, Na_v_1.7	Expressed	Potential roles in mechanotransduction, endothelial permeability, and nitric oxide signaling; cardiac endothelial evidence remains limited [80]	NE	Expressed	Potential roles in vascular reactivity, proliferation, and migration in broader vascular literature [80]	Vascular remodeling/atherosclerosis proposed, not yet cardiac-cell-specific [80]	NE	NE	NE
Chloride	CLC	Expressed	Promotes Cl^−^ efflux, increases intracellular Ca^+^ concentration [81,82]	NE	Expressed	Cell volume homeostasis, cell proliferation, and apoptosis [83]	Hypertension-associated vascular diseases [83]	NE	NE	NE
	CaCC	Expressed	Promotes Cl^−^ efflux, increases intracellular Ca^+^ concentration [81,82]	NE	Expressed	Promotes Cl^−^ efflux, increases intracellular Ca^+^ concentration and SMC contraction [81,82]	NE	Expressed	Regulates local hemodynamics [81,82]	NE
Connexin	Cx37, Cx40, Cx43, Cx45	Expressed/functionally coupled	Transmit the cell membrane potential along the endothelium, modulating arteriolar tone and causing vasodilation [75,84,85]	NE	Expressed/functionally coupled	Regulate cellular function, vessel diameter, and blood flow [84,85]	NE	NE	NE	NE
Pannexin	Panx1	Expressed	Involved in ATP release and proinflammatory immune-cell recruitment [47]	Heart failure [47]	Expressed	Regulates cellular function, vessel diameter, and blood flow [84,85]	NE	NE	NE	NE
Calcium entry	Orai1/STIM1	Expressed/functionally plausible	Regulates endothelial Ca^2+^ signaling, barrier function, migration, proliferation, and angiogenic or inflammatory responses [4,49,73,75,82,86].	Vascular dysfunction and remodeling; cardiac microvascular disease relevance requires validation [4,49,75,82,86].	Expressed/functionally plausible	Supports vascular smooth-muscle Ca^2+^ signaling, proliferation, contraction, and remodeling [4,49,73,75,82,86].	Hypertension and vascular remodeling; cell-specific cardiac evidence remains incomplete [4,73,75,82].	NE	NE	NE

**Table 3:** Summary of ion channel expression in immune cells. NE, not established.

Type	Name	Cell Type with Evidence	Function	Disease
Mechanosensitive	PIEZO1	Macrophages	Regulating inflammatory activation, angiogenesis, and tissue repair [88,89,90,91,92]	Aortic valve stenosis, angiogenesis [2,90,91,92]
	K_ATP_	Macrophages	Modulating macrophage polarization, suppressing inflammatory responses, and promoting angiogenesis [91,97]	Angiogenesis [91,97]
TRP	TRPC1, TRPC3	T cells	Regulate Ca^+^ influx and cell proliferation [98,99]	NE
	TRPC2, TRPM1, TRPM5, TRPM6, TRPM7, TRPV3	T cells	NE	NE
	TRPC5	T cells	Mediate Ca^+^ influx [100]	NE
	TRPC6	T cells	Mediate T cell apoptosis [101]	NE
	TRPM2	T cells	Induces Ca^+^ influx, regulate T cell proliferation, and regulate pro-inflammatory cytokine production [98,102]	NE
	TRPM4	Macrophages and T cells	Promotes inflammatory-cell infiltration, macrophage proliferation, and inflammatory gene expression [91]	Myocardial infarction [91]
	TRPV1	T cells, macrophages, monocytes, dendritic cells	Regulates inflammatory signaling, vasodilator neuropeptide release, angiogenesis, and immune-cell activation [91,103,104,105,106]	Atherosclerosis [107]
	TRPV2	Macrophages and T cells	Enhances macrophage phagocytic activity and promotes macrophage recruitment [91,103]	Myocardial infarction [91,103]
	TRPV4	T cells, macrophages, neutrophils	Regulates macrophage-cardiomyocyte interactions, angiogenesis, macrophage infiltration, VEGFA expression, and neutrophil/T-cell activation [91,98,108,109,110]	Dilated cardiomyopathy [109]
	TRPV5/6	T cells	Involved in Ca^+^ entry and cell cycle [111]	NE
	TRPA1	Macrophages and T cells	NE	NE
	TRPML1/2	B cells	NE	NE
Acid-sensing	ASIC1	Macrophages and dendritic cells	Regulating phagocytosis rate, release of anti-inflammatory cytokines, and lipophagy [112,113,114]	Atherosclerosis [114]
	ASIC2	Dendritic cells	T cell activation and proliferation [112]	NE
	ASIC3	Macrophages and dendritic cells	T cell activation and proliferation [112]	NE
Cation selective: K^+^	Kv1.3	Macrophages and T cells	Promotes immune cell migration, T cell proliferation, and cytokine production [115,116]	Atherosclerosis/acute coronary syndrome-associated immune activation [116]
	Kv1.5	Macrophages	Promotes macrophage migration [116]	NE
	KCa2.1/SK1, KCa2.2/SK2, KCa2.3/SK3	Macrophages	Regulate macrophage differentiation and activation [117]	NE
	K_Ca_3.1	Macrophages and T cells	Promotes immune-cell migration, T-cell proliferation, cytokine production, and proinflammatory macrophage polarization [91,115,118]	Post-MI arrhythmogenic inflammation and atherosclerosis [91,119,120]
	Kir2.1	Macrophages	Macrophage proliferation and activation [118]	NE
Cation selective: Na^+^	Na_v_1.2, Na_v_1.3	Macrophages	Evidence limited; sodium-channel inhibitor studies support immune modulation but isoform specificity remains incomplete [91,121]	Atherosclerosis/vascular inflammation; cardiac remodeling evidence remains indirect [121]
	Na_v_1.4, Na_v_1.9	Macrophages and monocytes	Regulating macrophage polarization [121]	Atherosclerosis/vascular inflammation; cardiac remodeling evidence remains indirect [121]
	Na_v_1.5	Macrophages and T cells	Influencing cardiomyocyte electrophysiology [122], and T cell development and activation [123]	Arrhythmia [122]
	Na_v_1.6, Na_v_1.7	T cells	Lymphocyte development, migration, and inflammatory responses inferred from T-cell Na_v_-channel literature [123]	NE
Chloride	CaCC	T cells	Maintaining T cell receptor activation and cytokine secretion [124]	NE
	VRAC	T cells	Regulating cell proliferation, migration, and immune synapse formation [124,125]	NE
	CLIC1	Macrophages and T cells	Regulates proinflammatory signaling in macrophages/T cells; direct cardiac immune-cell evidence remains limited [91,124]	Chronic inflammatory diseases [91]
	CLIC3	Macrophages and T cells	Promotes macrophage polarization; direct cardiac immune-cell evidence remains limited [124,126]	NE
	CLIC4	Macrophages and T cells	Regulates proinflammatory activators; direct cardiac immune-cell evidence remains limited [124,127]	NE
Connexin	Cx37	Macrophages and monocytes	Athero-protective by regulating leukocyte recruitment and adhesion to the endothelium [128,129]	Atherosclerosis [128,129]
	Cx43	Macrophages and T cells	Enabling synchronous depolarization of the negative resting membrane potential to promote signaling propagation [130]. In T cells, it contributes to immune synapse Formation, calcium wave propagation, and cytokine signaling [131]	AV conduction delay, ventricular arrhythmias [130]
Pannexin	Panx1	Macrophages and T cells	T cell migration, macrophage activation, and regulation of adaptive immune responses [132]	Immune activation/inflammatory recruitment; direct cardiac disease role remains to be established [132]
Calcium entry	Orai1/STIM1; CRAC channels	Macrophages, T cells, and other immune cells	Central regulators of Ca^2+^-dependent lymphocyte activation, cytokine production, macrophage inflammatory programs, and immune-cell migration [48].	Inflammation, atherosclerosis, and post-injury immune activation; direct cardiac non-myocyte targeting remains to be established [48].

**Table 4:** Summary of ion channel expression in adipocytes. NE, not established.

Type	Name	Function	Disease Association
Mechanosensitive	PIEZO1	Adipocyte differentiation, adipose remodeling, and systemic metabolic regulation [6]	Adipose inflammation, obesity [6]
TRP	TRPV1	Reduces adipose inflammation, increases insulin sensitivity to regulate glucose and fat metabolism [68]	Atherosclerosis/metabolic inflammation in broader TRPV1 literature; direct cardiac adipocyte disease role NE [68]
Acid-sensing	ASIC1	NE	Obesity, insulin resistance, and hypertension [135,136]
Cation-selective: K^+^	Kv1.3, Kv1.5, Kv1.6, Kv4.2, Kv4.3, KCa1.1, KCa2.1, KCa2.2, KCa2.3, KCa3.1, Kir2.1, Kir2.3	Adipogenesis, adipocyte maturation, membrane potential, and metabolic homeostasis in adipocyte literature [6]	Insulin resistance, obesity, altered adipokine secretion, and systemic metabolic dysfunction; cardiac adipocyte specificity remains NE [6]
Cation-selective: Na^+^	Na_v_1.2, Na_v_1.3, Na_v_1.4, Na_v_1.5, Na_v_1.6, Na_v_1.7, Na_v_1.9	Adipocyte differentiation and intracellular ion homeostasis in broader adipocyte literature [6]	Obesity-related inflammation, insulin resistance, and metabolic dysfunction; cardiac adipocyte specificity remains NE [6]
Chloride	CLIC1, CLIC3, CLIC4	Adipogenesis, cell volume regulation, lipid metabolism, and insulin signaling [125,137]	Obesity, adipose tissue inflammation, and insulin resistance; cardiac adipocyte specificity remains NE [138]

**Table 5:** Disease-centered summary of ion channels in cardiac non-excitable cells.

Disease Category	Key Non-Excitable Cells	Representative Channel Axes	Approach	Genetic or Small-Molecule Targeting Evidence
Fibrosis and adverse remodeling	Fibroblasts; macrophages; endothelial cells	TRPV4 [10,31]	Global/endothelial deletion; HC067047 antagonist	TRPV4 genetic deletion [154] and TRPV4 antagonist HC067047 [184] mitigate post-MI or diabetic cardiac fibrosis
		KATP [10,25,26,142,143,185,186]	Nicorandil/openers; risk-aware modulation	Nicorandil reduces AngII-induced fibroblast proliferation [185]; broader KATP modulation requires arrhythmia-risk awareness.
		PIEZO1 [10,15,16,17,19,20,21,22,23]	Yoda1 activation; gsmtx4 inhibition; genetic perturbation	Yoda1 activation [5] and PIEZO1 inhibition with gsmtx4 [56] support mechanistic modulation, but cardiac-fibrosis mitigation remains insufficiently established.
		TRPM7 [10,37,187], TRPC3/6 [31,33,34,63,149], TREK-1 [10,24,141], Cx43 [42,43,44,45], Panx1 [43,46,47,180]	Genetic deletion; channel blockers; gap-junction/hemichannel modulation	Evidence supports disease mechanisms, but cell-selective therapeutic tests remain incomplete.
Heart failure and pressure overload	Fibroblasts; endothelial cells; pericytes; immune cells; adipocytes	TRP channels [10,31,33,34,35,37,38,39,63,73,98,99,100,101,149,150,158,187,188,189,190]	Endothelial-specific TRPV4 deletion; TAC pressure-overload model; RNA-seq/YAP-VEGFR2 angiogenesis readouts; AAV9-shcd36 metabolic intervention	Endothelial TRPV4 deletion [191] protects pressure-overloaded hearts; partial AAV9-shcd36 knockdown [192] prevents TAC-induced hypertrophy and dysfunction
		KATP [10,25,26,142,143,185,186]	Nicorandil/openers; risk-aware modulation	KATP activation is cardioprotective in I/R [142,143], but KATP/pinacidil can be proarrhythmic [10,28,144].
		PIEZO1 [9,10], TREK-1 [10,24,141], Panx1 [47], connexins [42,43,44,45,46,128,152,179]	Conditional deletion; channel blockade; connexin/pannexin modulation	Mechanistic evidence links these axes to pressure-overload and heart-failure remodeling, but therapeutic direction is context-dependent [10,24,43,47,141].
Arrhythmogenesis and conduction remodeling	Fibroblasts; macrophages; endothelial cells	Cx43 [91,130], Panx1 [43,47]	Connexin hemichannel/Panx1 blockade; mimetic peptide or channel-inhibitor testing; macrophage Cx43 and paracrine-coupling models	Cx43 and Panx1 are proposed antiarrhythmic targets; macrophage Cx43 supports arrhythmia mechanisms [43,130].
		Kv/KCa channels [91,116,117,119,120,169,170,171]	TRAM-34/clotrimazole; KCa3.1 silencing;	Macrophage KCa3.1 supports post-MI arrhythmogenic inflammation [119].
		KATP [10,28,144]	Nicorandil/openers; risk-aware modulation	KATP/pinacidil opening can promote arrhythmogenic electrical heterogeneity [10,28,144]
		Sodium channels [80,91,121,122,123,175]	Sodium-channel inhibition	Sodium-channel inhibition [175] reduces monocyte/macrophage inflammatory injury
Ischemia–reperfusion injury and infarct healing	Endothelial cells; macrophages; fibroblasts; smooth muscle cells	ASICs [40,74,112,113,114,160,161,162,163]	Small-molecule inhibition	ASIC1a inhibition [160] recovers heart function after I/R
		Sodium channels [121,122,123,175]	Sodium-channel inhibition	Sodium-channel inhibition [175] reduces monocyte/macrophage inflammatory injury
		Panx1 [47,181]	Genetic deletion; nanobody blockade	Panx1 deletion [47,181] or Panx1 nanobody blockade [193] improves I/R outcomes
		KATP [10,25,26,142,143,185,186]	Nicorandil/openers; risk-aware modulation	KATP activation supports cardioprotection [142,143]
		Cx43 [42,43,44,45], PIEZO1 [88,89,90,91,92,93,94]	Cell-selective perturbation; mechanoinflammatory pathway testing	These axes are implicated in infarct healing and immune-fibroblast crosstalk, but direct therapeutic evidence is still limited.
Vascular dysfunction, hypertension, and microvascular disease	Endothelial cells; smooth muscle cells; pericytes	PIEZO1 [4,8,54,55,87]	Yoda1 activation; gsmtx4 inhibition; genetic perturbation	Smooth-muscle PIEZO1 loss of function implicates PIEZO1 in hypertension-dependent arterial remodeling [54]; endothelial PIEZO1/TRPV4-ADAM signaling supports vascular mechano-transduction [4].
		KATP [27,29,30,57,58,59,60,61,62,75,145,146,147], Kv [75,76,166,167], KCa1.1/BK [62,164,165], KCa2.x/KCa3.1 (SK/IK) [75,77,151]	KATP opener/blocker testing; Kv/BK/SK/IK modulation; pressure-myography and microvascular reactivity assays	Vascular KATP/Kv/KCa modulation [27,29,30,57,58,59,60,61,62,75,145,146,147] is pharmacologically tractable for vascular tone
		Ano1/TMEM16A [75,81,82,177]	Vascular Tmem16a/Ano1 conditional disruption; CaCC inhibition; smooth-muscle and pericyte contractility assays	Ano1/TMEM16A disruption [81] lowers blood pressure
		Cx37/40/43 [84,85,128,129,152]	Connexin/hemichannel blockade; myoendothelial coupling assays; endothelial-smooth muscle electrical communication studies	Connexin/hemichannel blockade [152,182] can reduce vasoconstriction
		TRPV4 [3,4,73]	Endothelial TRPV4 modulation; vascular-bed-specific testing	Endothelial TRPV4 supports vasodilation and vascular mechano-transduction [3,4,73]; disease direction depends on vascular bed and disease context.
Atherosclerosis and vascular inflammation	Macrophages; endothelial cells; smooth muscle cells; lymphocytes	PIEZO1 [56]	Gsmtx4-mediated PIEZO1 inhibition; disturbed-flow and partial-carotid-ligation atherosclerosis models; endothelial inflammation readouts	PIEZO1 inhibition with gsmtx4 [56] delays plaque progression
		KCa3.1 [91,120]	TRAM-34/clotrimazole; KCa3.1 silencing;	KCa3.1 blockers TRAM-34/clotrimazole and KCa3.1 silencing reduce atherosclerosis [157]; sodium-channel inhibition reduces monocyte/macrophage inflammatory injury [121].
		Cx37 [129]	Gja4/Cx37 genetic loss-of-function; adoptive monocyte-transfer assays; hemichannel ATP-release and leukocyte-adhesion testing	Cx37 reduces macrophage-driven atherosclerosis and is genetically protective [129].
		Kv1.3/Kv1.5 [119,120,169,170], sodium channels [121,122,123,175], Panx1 [132,183], ASICs [40,74,112,113,114,160,161,162,163]	Kv/K_Ca_ blockade; sodium-channel inhibition; purinergic/ASIC modulation	Kv1.3/Kv1.5 and sodium-channel studies support macrophage/lymphocyte inflammatory mechanisms, but direct cardiac translation remains limited [116,120,121,169,170].
Obesity-associated cardiometabolic remodeling	Adipocytes; endothelial cells; macrophages	PIEZO1 [6,133,134], TRPV channels [6], chloride channels [124,125,137], connexins/pan [119,120,169,170] nexins [43,47]	Transcriptomic nomination; metabolic intervention; adipocyte-specific validation	Evidence remains mostly indirect; CD36 knockdown supports metabolic-target modulation in pressure-overload remodeling [192], but adipocyte-specific channel targeting needs validation [6].
Fibrosis, vascular remodeling, endothelial dysfunction, and inflammation	Cardiac fibroblasts; endothelial cells; vascular smooth-muscle cells; macrophages; lymphocytes	STIM1/Orai1-dependent store-operated Ca^2+^ entry [4,48,49,51,73,75,86]	Patch clamp or Ca^2+^ imaging; pharmacological or genetic perturbation; disease-model validation; human tissue or omics support where available	STIM/Orai-mediated Ca^2+^ entry has been linked to fibroblast activation and matrix remodeling, endothelial dysfunction and angiogenic/inflammatory signaling, vascular smooth-muscle proliferation or migration, and immune-cell activation. In the heart, the strongest relevance is to fibrosis, adverse remodeling, ischemia/reperfusion-associated inflammation, and vascular remodeling, although cell-selective cardiac therapeutic evidence remains incomplete [4,48,49,51,73,75,86].

## Data Availability

No new data were created or analyzed in this study. Data sharing is not applicable to this article.
